# Detection of Cyanotoxins in Algae Dietary Supplements

**DOI:** 10.3390/toxins9030076

**Published:** 2017-02-25

**Authors:** Audrey Roy-Lachapelle, Morgan Solliec, Maryse F. Bouchard, Sébastien Sauvé

**Affiliations:** 1 Department of Chemistry, Université de Montréal, Montréal, QC H3T 1J4, Canada; a.roy.lachapelle@umontreal.ca (A.R.-L.); morgan.solliec@umontreal.ca (M.S.); 2 Department of Environmental and Occupational Health, Université de Montréal, Montréal, QC H3T 1A8, Canada; maryse.bouchard@umontreal.ca

**Keywords:** cyanotoxins, cyanobacteria, dietary supplements, LC-HRMS, LDTD, microcystins, anatoxin-a, cylindrospermopsin, BMAA, saxitoxin

## Abstract

Algae dietary supplements are marketed worldwide as natural health products. Although their proprieties have been claimed as beneficial to improve overall health, there have been several previous reports of contamination by cyanotoxins. These products generally contain non-toxic cyanobacteria, but the methods of cultivation in natural waters without appropriate quality controls allow contamination by toxin producer species present in the natural environment. In this study, we investigated the presence of total microcystins, seven individual microcystins (RR, YR, LR, LA, LY, LW, LF), anatoxin-a, dihydroanatoxin-a, epoxyanatoxin-a, cylindrospermopsin, saxitoxin, and β-methylamino-l-alanine in 18 different commercially available products containing *Spirulina* or *Aphanizomenon flos-aquae*. Total microcystins analysis was accomplished using a Lemieux oxidation and a chemical derivatization using dansyl chloride was needed for the simultaneous analysis of cylindrospermopsin, saxitoxin, and β-methylamino-l-alanine. Moreover, the use of laser diode thermal desorption (LDTD) and ultra-high performance liquid chromatography (UHPLC) both coupled to high resolution mass spectrometry (HRMS) enabled high performance detection and quantitation. Out of the 18 products analyzed, 8 contained some cyanotoxins at levels exceeding the tolerable daily intake values. The presence of cyanotoxins in these algal dietary supplements reinforces the need for a better quality control as well as consumer’s awareness on the potential risks associated with the consumption of these supplements.

## 1. Introduction

Cyanobacteria (CB), formally called blue-green algae, are omnipresent in natural water sources and cause increased concern for public health [1]. Although they are mostly innocuous, a significant proportion of cyanobacterial species are harmful due to toxin production [2]. Cyanotoxins have several modes of action including: hepatotoxicity, affecting mainly the liver; neurotoxicity, acting on the nerve cells; cytotoxicity, affecting the cells and potentially carcinogenic; and dermatotoxicity, acting as irritant [3]. The production of massive cyanobacterial blooms can be caused by eutrophic conditions in the presence of heat, light, shallow waters, and nutrients [1,3]. Moreover, intense agriculture activities, and more recently, climate changes have been the principal triggers of massive cyanobacterial blooms [4,5]. The production of cyanobacteria, as well as their cyanotoxins, is almost impossible to predict precisely [6]. The nature of the producing cells and toxins, the localization of proliferation, and the duration of the bloom at a specific location are hard to characterize, which makes their management difficult.

CB have been commercialized as dietary supplements and recently gained popularity for their alleged beneficial effects on health for adults and children, including an increase in energy, better mood, antioxidant proprieties, and anti-cancer effects [7,8,9]. Our investigation included two types of CB supplements: *Spirulina*, which is now recognized by two species named *Arthrospira platensis* and *Arthrospira maxima,* the latter being found worldwide and are considered innocuous [10]; and non-toxic *Aphanizomenon flos-aquae* is also used from blooms to produce food supplements and has been primarily harvested from Upper Klamath Lake in Oregon, USA. However, toxic strains of *A. flos-aquae* may be found occasionally in some supplements, and they have been reported to produce paralytic shellfish poisons and anatoxin-a [11]. Moreover, despite some quality control as described by Carmichael et al. [10], other CB species can accidentally contaminate the products since the production is made in the natural environment. Indeed, the presence of microcystins and alkaloid cyanotoxins producers have been found in supplements [10,12,13,14]. Yet, most dietary supplements suffer from less strict quality controls since they are categorized as foodstuff products and not as pharmaceuticals [15]. Moreover, they are marketed internationally, sold over-the-counter, and easily accessible via the Internet without any guarantee of proper quality controls. Previous studies showed the presence of microcystins at concentration levels exceeding the proposed World Health Organization’s (WHO) tolerable daily intake (TDI) of 0.04 µg∙kg^−1^ body weight for an adult [16,17,18,19,20]. This TDI is an estimate of the amount of microcystins that can be ingested daily over a lifetime without appreciable health risk. Anatoxin-a and its congeners have been reported in different brands containing both *Spirulina* and *A. flos-aquae* [13,17]. Moreover, some strains of *A. flos-aquae* are known to produce saxitoxins and β-methylamino-l-alanine, the presence of the latter having been confirmed in CB dietary supplements [21,22,23,24,25,26].

In this paper, we aim to analyze different brands of CB dietary supplements to screen for the presence of different cyanotoxins: microcystins (MCs), anatoxin-a (ANA-a), cylindrospermopsin (CYN), saxitoxin (STX), and β-methylamino-l-alanine (BMAA). The use of different analytical strategies coupled with high resolution mass spectrometry (HRMS) detection allows us to confirm the presence of different cyanotoxins which could not be analysed in a single analytical run due to their structure specificity. The structural identification and compound profiles were obtained using the Q-Exactive orbital ion trap, which enabled selective detection for all targeted compounds, both with and without the use of certified standard materials. For this purpose, we used different analytical approaches previously developed in our laboratory including laser diode thermal desorption (LDTD) for a fast screening of total MCs and ANA-a (approximately 10 s per sample) while eliminating a chromatographic separation step [27,28,29]. More specifically, Lemieux oxidation using potassium permanganate and sodium periodate allowed the recovery of the 2-methyl-3-methoxy-4-phenylbutyric acid (MMPB) moiety common to all MCs which includes over 100 known congeners [30]. Samples were also submitted to ultra-high performance liquid chromatography-heated electrospray (UHPLC-HESI) coupled to HRMS, which were previously developed and ultimately adapted for this project [31]. The MC congeners present in the samples were then identified and quantified and results were compared with total MCs quantification results. Transformation products of ANA-a were qualitatively searched including dihydroanatoxin-a (DH-ANA-a) and epoxyanatoxin-a (E-ANA-a) and they were semi-quantified using ANA-a as a reference standard [32]. Finally, the analysis of CYN, STX, and BMAA was possible with a derivatization step using dansyl chloride (DNS) which was also previously developed [33].

Upon long term consumption, the presence of undesirable and harmful toxins in dietary supplements would lead to public health concerns as they are widely available, labeled as safe products, and even promoted as beneficial for health. By screening a large variety of cyanotoxins in these supplements, we aim to assess their toxic potential.

## 2. Results and Discussion

### 2.1. Multi-Toxins Analysis and Validation

Different analytical approaches have been used to determine the presence of several cyanotoxins in *Spirulina* and *A. flos-aquae* dietary supplements available worldwide in stores or via the Internet [11,12,13,17,34,35,36]. Analytical methods for the analysis of cyanotoxins, until now, cannot encompass the presence of a multitude of congeners and their diversity in terms of physicochemical properties. Using previously developed analytical methods for different cyanotoxin families, we were able to analyse total MCs via the MMPB moiety and compare results to multi MCs detection using seven standards. We were also able to compare results using ultra-fast detection of ANA-a using the LDTD-APCI-HRMS to UHPLC-HESI-HRMS method. The detection of CYN, STX, and BMAA was possible using DNS derivatization prior to UHPLC-HESI-HRMS analysis. Finally, transformation products of ANA-a were evaluated and confirmed using HRMS detection and were semi-quantified. To evaluate the validation parameters from the different analytical methods, *Chlorella* based dietary supplement products were used as relevant matrix material, thus as method blanks. Indeed, *Chlorella* is used and sold similarly as other CB dietary supplements, only, instead of being a cyanobacterium, this genus is a unicellular green algae. Nevertheless, matrix blanks were evaluated in every analysis to ensure the absence of false positives.

#### 2.1.1. Evaluation of Extraction Methods

Different extraction methods were employed depending on the targeted cyanotoxins and the analytical methods. The extraction recoveries (Table 1) and matrix effects (Table S1) were evaluated at two concentration levels for all target cyanotoxins in triplicate at two levels of concentrations. The extraction recovery of total MCs was evaluated using a Lemieux oxidation protocol previously developed and validated in our laboratory and other research groups [28,29,37,38]. The extraction recoveries, including oxidation reaction and SPE extraction, were 79% and 85% and signal recoveries from matrix effects were 85% and 87%. The analysis of ANA-a using the LDTD-APCI-HRMS method resulted in extraction recoveries of 95% and 92% and signal recoveries from matrix effects of 90% and 92%. For the analysis of the different MCs and ANA-a, results showed extraction recoveries between 85% and 96% and signal recovery from matrix effects between 91% and 112%, respectively. Finally, for the derivatization and SPE extraction steps combined of CYN, STX, and BMAA, extraction recoveries ranged from 83% to 97% and signal recoveries from matrix effect ranged from 94% to 109%. Globally, the different extraction steps showed acceptable recoveries while eliminating substantial background signals which could otherwise contribute to enhance matrix effects. It was acceptable with no significant signal enhancement or loss. The only exceptions came from the analysis of total MCs via the MMPB moiety. Lower extraction recoveries were obtained due to the complexity of the extraction steps necessary for obtaining the MMPB moiety, but compared to previous studies, these values are deemed acceptable [37,38]. Also, slight signal suppression was observed, which can be due to the presence of residual salts from the oxidation reagents, which can interfere with the LDTD desorption [28].

#### 2.1.2. Methods Validation

Method validation was done by evaluating linearity, precision (intraday and interday), accuracy, method detection (MDLs) and quantification limits (MQLs) in *Chlorella* samples used as matrix blanks (Table 1). The calibration curves of the spiked cyanotoxins showed good linearity with *R*^2^ ≥ 0.9985 using the corresponding internal standards. The precision and accuracy (% bias) of the methods were determined at two concentration levels by analysing replicates of spiked blank matrix samples in triplicate. The precision and the accuracy for all analytes were deemed suitable, ranging from 1% to 15% and between 2 and 12, respectively. The MDLs and MQLs values ranged from 0.01 to 0.3 μg∙g^−1^ and 0.03 to 0.8 μg∙g^−1^, respectively. The evaluated MDLs and MQLs of the present developed method were comparable to previous developed methods for the analysis of cyanotoxins in freeze-dried cyanobacterial cells [39,40]. In comparison, UHPLC-HESI-HRMS methods offered better results in terms of MDLs and MQLs, which was expected. However, the results obtained for total MCs via MMPB and ANA-a were substantially lower than provisional TDI guidelines being approximately 2.4 µg of MC-LReq and 6 µg daily for an adult weighing 60 kg compared to the MDL being 0.2 and 0.3 µg∙g^−1^, respectively [20,41]. Considering this, the LDTD-APCI-HRMS analytical apparatus given by previous methods and this paper can be a great tool for faster screening of the target cyanotoxins as demonstrated.

### 2.2. Toxins Quantification in CB Dietary Supplements

**Figure 1:**
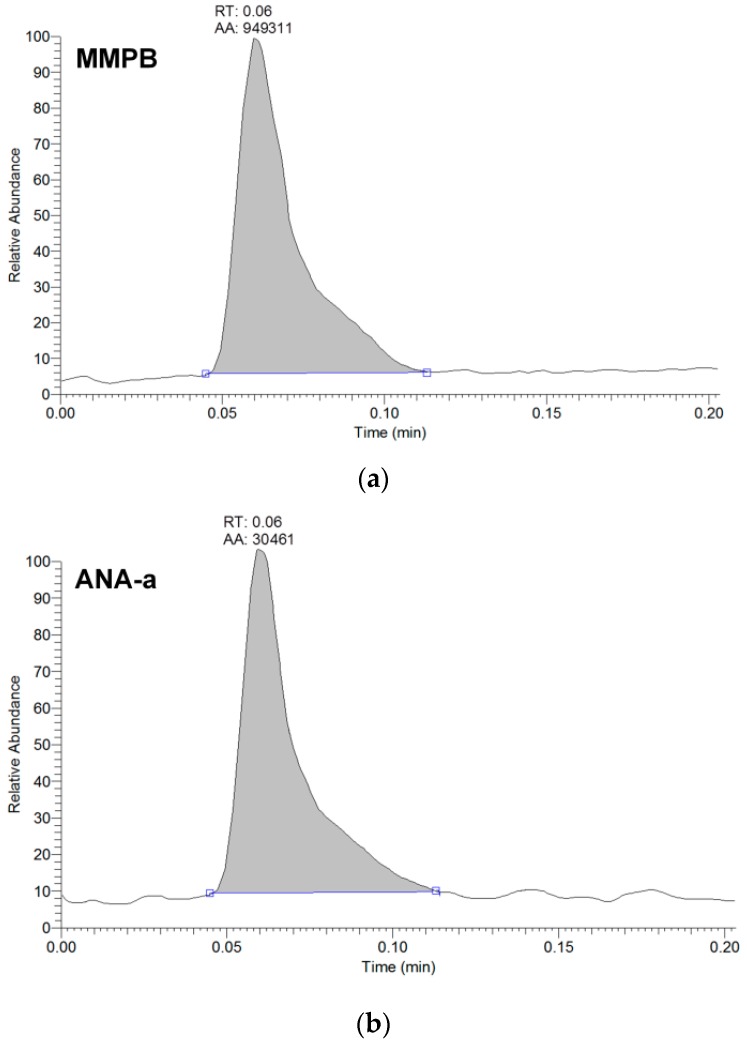
Example of peak shape from the analysis of brand #17 using LDTD-APCI-HRMS detecting (**a**) MMPB for total microcystins analysis, and (**b**) anatoxin-a (ANA-a). The analytes are represented by the integrated peaks with corresponding retention times (RT) and area (AA).

**Figure 2:**
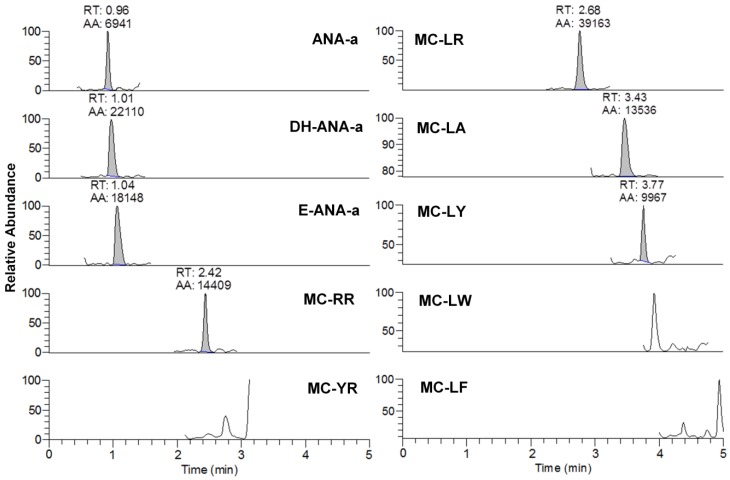
Example of chromatogram from the analysis of brand #15 using DNS derivatization and UHPLC-HESI-HRMS analysis for anatoxin-a (ANA-a), dihydroanatoxin-a (DH-ANA-a), epoxyanatoxin-a (E-ANA-a), and seven microcystins (RR, YR, LR, LA, LY, LY, LW, and LF). The analytes are represented by the integrated peaks with corresponding retention times (RT) and area (AA).

**Figure 3:**
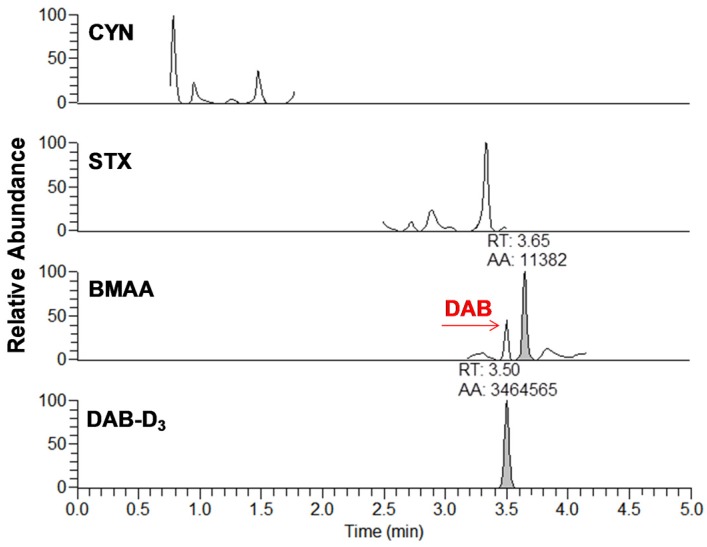
Example of chromatogram from the analysis of brand #15 using DNS derivatization and UHPLC-HESI-HRMS analysis for cylindrospermopsin (CYN), saxitoxin (STX), β-methylamino-l-alanine (BMAA), and diaminobutyric acid-D_3_ (DAB-D_3_). The analytes are represented by the integrated peaks with corresponding retention times (RT) and area (AA).

From the 18 CB dietary products tested, fourteen contained *Spirulina* and four contained *A. flos-aquae*. They were analysed on a mass basis (as µg of cyanotoxins per g of dry weighed CB) and results are presented in Table S2. Three brands out of the 14 *Spirulina* products contained amounts of total MCs varying between 0.25 and 0.84 µg∙g^−1^. In comparison, the concentrations obtained using the sum of individual MC standards varied between 0.01 and 0.63 µg∙g^−1^. As for the *A. flos-aquae*-based brands, amounts of total MCs were substantially higher with results varying between 0.8 and 8.2 µg∙g^−1^ using the Adda oxidation method. In comparison, by using the sums of MCs standards, the concentrations varied between 0.52 and 5.8 µg∙g^−1^. As seen in previous studies, there is a possibility of underestimation when total MC is not taken into account since only a limited number of MC variants are available as standards (approximately 10%). In our case, the presence of unseen variants and congener isomers is probable considering the results obtained. Two methods were used to quantify ANA-a, the first using the LDTD-APCI-HRMS for faster screening and the second using UHPLC-HESI-HRMS to include the toxin in a multi-toxins analytical method. Results showed the presence of ANA-a in two *A. flos-aquae* CB dietary supplements. In one of these samples, similar amounts were detected using the two analytical methods, i.e., 0.44 µg∙g^−1^ of ANA-a using the LDTD-APCI-HRMS and 0.40 µg∙g^−1^ using the UHPLC-HESI-HRMS. In the other sample, only the second analytical method detected ANA-a, with an obtained amount of 0.15 µg∙g^−1^, which is explained by the MDL of the two different methods. An example to illustrate the LDTD peak shape of MMPB and ANA-a is presented in Figure 1 from brand #17. Figure 2 illustrates the chromatographic separation of ANA-a and the individual MCs from brand #15. Finally, CYN and STX were not detected in any samples, but two samples of *A. flos-aquae* contained BMAA with amounts of 0.04 and 0.55 µg∙g^−1^. An example of the chromatographic separation is shown in Figure 3 from brand #15 including a clear separation of BMAA and its isobaric isomer 2,3-diaminobutyric acid (DAB). Globally, *Spirulina*-based brands contained less cyanotoxins than *A. flos-aquae* based brands and this phenomenon was previously observed [42]. Normally, *Spirulina* does not have the capacity to produce cyanotoxins [10]. Moreover, their harvest requires specific conditions (pH 9–10) which are not suitable for the efflorescence of a large number of CB [11]. However, it was previously found that *Arthospira*, a potential ANA-a producer, can appear in the harvest of *Spirulina* and can be missed during quality controls, due to their similar morphology [14,43]. The presence of other toxic CB such as *Microcystis* has also been documented [44]. As for *A. flos-aquae*, all our selected brands showed the presence of cyanotoxins, which is in concordance with many studies [12,13,19]. Such samples mostly contain MCs, but previous analysis showed that alkaloid cyanotoxins can also be found in *A. flos-aquae* culture, therefore in CB dietary supplements. BMAA was found in two samples containing *A. flos-aquae*. BMAA can cause neurodegeneration in animal models, and is suspected to be linked to neurodegenerative diseases [45,46]. Thus, its mere presence in a food supplement represents a potential concern for public health.

### 2.3. Transformation Products ANA-a

**Figure 4:**
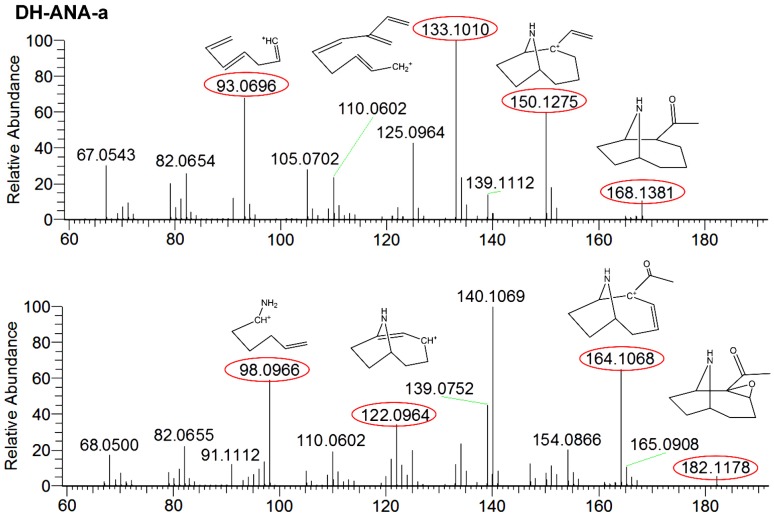
Fragmentation mass spectra of dihydroanatoxin-a (DH-ANA-a) with targeted product ions *m*/*z* 168 > 93, 133, and 150 and epoxyanatoxin-a (E-ANA-a) with targeted product ions *m*/*z* 182 > 98, 122, and 164.

ANA-a is a labile compound which is easily transformed under natural conditions, including alkaline pH and UV light. However, it can be transformed into less toxic and more stable compounds, which includes DH-ANA-a and E-ANA-a. Their presence was monitored to assess the possibility of ANA-a production in a CB harvest, which can add a possible presence of toxic CB in the cultures that would not be detectable in the products. Due to the lack of certified standards, these compounds were searched and confirmed using HRMS detection and they were semi-quantified using the ANA-a standard for calibration. DH-ANA-a was found in three samples at concentrations varying between 0.34 and 7.2 µg∙g^−1^ and E-ANA-a was found in four samples at concentrations between 0.07 and 2.7 µg∙g^−1^. These concentrations, even if approximate, were substantially higher than concentrations obtained for ANA-a. Similarly to our results, Draisci et al. [17], found transformation products of ANA-a without finding the parent molecule. This suggested a potential health hazard associated with CB dietary supplement consumption. To ensure the identification of the compounds, comparisons of fragmentation patterns, which are shown in Figure 4, were done using the Mass Frontier^TM^ software. The obtained results confirmed their structure identification, therefore supporting their presence in samples. For DH-ANA-a, the targeted product ions for identification were *m/z* 168.1381 > 96.0396, 133.1010, and 150.1275. For E-ANA-a, the product ions were *m/z* 182.1178 > 98.0966, 122.0964, and 164.1068. These masses are in accordance with the results presented in previous studies which were obtained using reference standards and LC-MS/MS detection [16,47]. Moreover, to ensure proper identification, the structures were searched via the Mass Frontier^TM^ software using their exact mass and are illustrated in Figure 4. Indeed, we found plausible structures for each of the masses used for identification with mass errors equal or below 3 ppm, thus confirming the presence of DH-ANA-a and E-ANA-a in the samples.

### 2.4. Risk Assessment

Various TDI guidelines have been proposed by many institutions for the intake of cyanotoxins in order to prevent toxic effects. The most recognized value was established by the WHO in the form of a TDI guideline of 0.04 µg∙kg^−1^ body weight for MCs. This value corresponds to an intake of 2.4 µg of MCs per day for an adult weighing 60 kg [20]. So far, the WHO has not established guidelines for cyanotoxins other than MCs. The Oregon Health Authority (OHA) Public Health Division have established guidelines for additional toxins as part of the implementation of a monitoring program for the presence of cyanotoxins [41]. They developed TDI values for ANA-a (6 µg), CYN (1.8 µg), MCs (3 µg), and STX (3 µg). Our results indicate that the consumption of these ‘health products’ might be associated with health risks because of the presence of cyanotoxins in several CB products. Table 2 presents the results on the quantity of cyanotoxins ingested according to TDI, when applicable, for each brand. Out of the 18 brands, 4 contained cyanotoxins at levels exceeding TDI values. According to our results, these brands showed amounts of MCs exceeding the recommended TDI for adults (exceeding TDI by up to 683 % for total MCs).

The TDI values are for adults weighing 60 kg and therefore should be reduced for children. For example, the intake for a child weighing 30 kg (average for a 10-year-old) should not exceed half of the TDI values established for an adult. In addition to the four brands exceeding the TDI for total MCs for adults, another brand had a total MCs value of 0.8 µg, which is equivalent to the TDI for child weighing 20 kg (this product that did not warn against use for children). In our study, a third of the products analyzed had warnings against consumption by children (Table 3). Yet, CB supplementation has been suggested as an alternative (natural) therapy for children with attention deficit/hyperactivity disorder (ADHD) [48]. Some of these products have also been presented as providing health benefits to pregnant women [49].

In addition, BMAA was found in two brands, although at low concentrations. The lack of toxicological data and guidelines does not allow the determination of the risk associated to chronic exposure of this compound. However, BMAA has been associated with neurodegenerative diseases [45,46], and it would seem appropriate to establish a preventive monitoring of this toxin including in CB dietary supplements. Furthermore, Andersson, M. et al. [50] have recently shown the possibility that mothers’ milk could be a source of exposure for BMAA in human infants. This would be due to a low metabolic (or even non-metabolic) elimination of BMAA in addition to its transportation as a free amino acid to rat milk and suckling pups. Breastfed infants are therefore put at risk, considering that not a majority of brands contraindicate their products for breastfeeding women. Finally, ANA-a or its transformation compounds, DH-ANA-a and E-ANA-a, were found in six different samples. This suggests the presence of alkaloid cyanotoxins producers in the CB cultures, both in *Spirulina* and *A. flos-aquae* harvests. The level of risk appears low in our study since the estimated intake in the two samples where it was detected was much lower than the TDI. However, our sample size was limited, thus other products available commercially might contain larger levels of these cyanotoxins.

Published reports raise concerns for risks of intoxication and liver injuries caused by a chronic consumption of CB dietary supplements [51,52]. Although subchronic cyanotoxin poisoning can lead to acute symptoms, the ingested doses must be at least 10-fold higher than the TDI guidelines [53]. Similarly, some cases of acute poisoning from MCs were previously documented, as well as exposition causing mild intoxication cases [54,55]. Although acute poisoning can lead to symptoms ranging from temporary gastrointestinal disorders to immediate liver failure, long term intoxication is known to increase the risk of liver cancer [56]. Indeed, some cyanotoxins have the propriety to bioaccumulate, especially MCs. These are known to covalently bind in liver tissues through protein phosphatase inhibition, inducing hepatic failure and tumor promotion [56,57]. This suggests the potential risk of a chronic consumption of CB dietary supplements contaminated by cyanotoxins.

In this study, we were able to screen many cyanotoxins to assess their toxic potential. Our results showed that some dietary products could be harmful upon long term consumption due to the undesirable presence of cyanotoxins. They are widely available, and labeled as safe products, and even beneficial for health. The presence of unwanted and harmful toxins shows the need for a more thorough monitoring of such products to evaluate the risks assessments and public health implications associated with the sale of such uncontrolled natural products. Finally, most guidelines for maximum intake of CB are established for a lifetime-based consumption. However, the United States Environmental Protection Agency recently proposed a 10-day health advisory for bottle-fed infants (0.3 µg∙L^−1^) and children of pre-school age (1.6 µg∙L^−1^) on the presence of MCs in drinking water [58]. This new advisory indicates the increasing concern of public health authorities for the risks associated with exposure to these toxins.

## 3. Conclusions

CB dietary supplements are marketed internationally, easily available, and advertised as beneficial for health. However, these products are sold with limited quality control, which could lead to potential toxic risks related to the contamination by cyanotoxins. We investigated the presence of several cyanotoxins in 18 brands of CB dietary supplements containing *Spirulina* and *Aphanizomenon flos-aquae* to assess the potential risks of chronic consumption from these products. We targeted total microcystins via the MMPB moiety, seven individual MCs, ANA-a, two of its transformation products, DH-ANA-a and E-ANA-a, cylindrospermopsin, saxitoxin and BMAA. These cyanotoxins were monitored using different analytical approaches including LDTD-APCI-HRMS, UHPLC-HESI-HRMS, and chemical derivatization in order to obtain a broad assessment of the presence of cyanotoxins. Results showed that 8 brands out of 18 contained some levels of cyanotoxins both in *Spirulina* and *A. flos-aquae* supplements. From these brands, four were contaminated with microcystins at levels exceeding the TDI values established by the WHO and the Oregon Health Division. Moreover, ANA-a and its transformation products were found in six brands which suggests the presence of alkaloid cyanotoxins producers species in CB cultures. Finally, low amounts of BMAA were found in two *A. flos-aquae* samples, supporting earlier reports that this toxin can appear in such dietary supplements [21,26]. This should be of concern for public health since dietary exposure to BMAA is suspected to be a potent neurotoxin. Finally, we do not want to emphasize the health risks associated with the specific supplement brands that we have analyzed. We rather pointed out that our data show the importance of better monitoring by the appropriate authorities of all algal-based food supplements and eventually prompt the adoption of health-based guidelines on the maximum intake to ensure the safety of these products.

## 4. Materials and Methods 

### 4.1. Chemicals, Reagents, and Stock Solutions

Detailed information on chemical standards, reagents, and solutions used in sample treatment, sample derivation and instrumental analysis are provided in the Supplementary Material (SI1).

### 4.2. Algal Dietary Supplement Samples

The samples of CB food supplements containing *Spirulina* and *A. flos-aquae* were obtained from several brands and different sources at different periods of time (Table 3). Some were purchased from commercial suppliers at different locations and others were purchased via the Internet. The cyanobacteria composition varied among samples and they included tablets, capsules, and powder. The CB dietary supplements were ground individually with a coffee grinder and 0.3 g was weighed from each powdered sample for subsequent extraction steps.

### 4.3. Sample Treatment Steps

#### 4.3.1. MMPB via LDTD-APCI-HRMS

For the analysis of total MCs via the MMPB moiety, the weighed samples were mixed in amber glass vials with 5 mL of the oxidation solution which consisted of KMnO_4_ (50 mM), NaIO_4_ (50 mM), and K_2_CO_3_ for a pH adjustment to 9 [28,29]. The oxidation duration was 2 h at room temperature and the reaction was quenched using a saturated solution of sodium bisulfite until the purple color in solution became colorless. The samples were acidified at pH ~ 2 using a solution of 10% sulfuric acid. Afterwards, these were filtered with 0.22 µm nylon membrane filters (Sterlitech Corporation, Kent, WA, USA). A solid phase extraction (SPE) step was employed for sample clean-up [28]. A styrene-divinylbenzene sorbent (Strata SDB-L) was used as the SPE cartridge from Phenomenex (Torrance, CA, USA) with 500 mg bed mass and a volume of 6 mL. A 12-position manifold from Phenomenex (Torrance, CA, USA) was used for the procedure. The SPE cartridges were conditioned with 5 mL of methanol (MeOH) followed by 5 mL of acidified water with 0.1% formic acid. The samples were loaded on the cartridge columns by gravity. The cartridges were then washed twice by adding 5 mL of water with 0.1% formic acid and containing 10% MeOH (*v*/*v*). The elution was performed using 5 mL of MeOH and the solvent was recovered into glass conical-bottom centrifuge tubes. The eluates were completely dried under a gentle stream of nitrogen at room temperature using a nine-port Reacti-Vap Pierce unit (Rockford, IL, USA). The dried fractions were then reconstituted in 500 µL of acetonitrile (ACN) and finally submitted to the LDTD-APCI-HRMS detection.

#### 4.3.2. ANA-a via LDTD-HRMS and MCs-ANA-a via UHPLC-HESI-HRMS

For the analysis of ANA-a via the LDTD-APCI-HRMS method and of MCs and ANA-a via the UHPLC-HESI-HRMS system, the weighed samples were mixed with 5 mL of acidified MeOH (formic acid, pH 2) in polyethylene conical-bottom centrifuge tubes [27,31]. The samples were homogenized for 10 min using ultrasonication and then they were submitted to centrifugation at 3500 rpm for 10 min. The supernatant was filtered using 0.22 µm nylon membrane filters and was transferred to a 15 mL conical-bottom glass centrifuge tube. Another 3 mL of acidified MeOH was added to the sample residues which were vortexed, centrifuged, and the supernatant was filtered and combined with the first suspension. The solvent was dried under a gentle stream of nitrogen at room temperature. For the analysis of ANA-a using the LDTD-APCI-HRMS, according to previous study, samples were reconstituted with 500 µL of a solution of H_2_O:MeOH (50:50 *v*/*v*) acidified with formic acid at pH ~ 2 and then they were directly submitted to analysis. For the analysis of MCs and ANA-a using the UHPLC-HESI-HRMS method, samples were reconstituted with 500 µL of acidified water with 0.1% formic acid and submitted for analysis.

#### 4.3.3. BMAA, CYN, and STX via UHPLC-HESI-HRMS

For the analysis of CYN, STX, and BMAA, the weighed samples were mixed in polypropylene conical-bottom centrifuge tubes with 5 mL of acidified water with citric acid (pH ~ 4) and were submitted to centrifugation at 3500 rpm for 10 min [33]. Thereafter, the supernatants were filtered using 0.22 µm nitrocellulose membrane filters (Millipore, Billerica, MA, USA) and then transferred into 15 mL polypropylene conical-bottom centrifuge tubes. Another 5 mL of acidified water was added to the sample residues to then be vortexed and centrifuged. The supernatant was filtered and combined with the first suspension. The combined solutions were submitted to a SPE clean-up step using a strong cation-exchange polymeric sorbent Strata-X-C cartridge (Phenomenex, Torrance, CA, USA) with 200 mg bed mass and a volume of 6 mL. The conditioning step was done with 5 mL of methanol (MeOH) followed by 5 mL of acidified water with citric acid (pH ~ 4). The acidified samples were then loaded on the cartridge columns by gravity. The cartridges were washed with 5 mL of acidified water (pH 4) containing 15% MeOH (*v*/*v*). Elution was performed with 5 mL of MeOH containing 3% NH_4_OH into conical-bottom polypropylene centrifuge tubes. The eluates were completely dried under a gentle stream of nitrogen at room temperature. The dried fractions were then reconstituted with the DNS reactive solution consisting of 250 µL of a Borax buffer (0.2 M, pH 9.5) and 250 µL of DNS in acetone (1 mg∙mL^−1^). The tubes were vortexed and placed in an Innova 4230 incubator shaker from New Brunswick Scientific (Edison, NJ, USA) at 60 °C for 10 min with agitation at 150 rpm. The samples were finally cooled at room temperature and were directly submitted to the UHPLC-HESI-HRMS analysis.

### 4.4. Analytical Detection

#### 4.4.1. LDTD Conditions

The analyte desorption was achieved with a T-960 LDTD-APCI ionization interface model and the instrument was controlled by LazSoft 4.0 software (Phytronix Technologies, Québec, QC, Canada) integrated with the Xcalibur 2.3 Software (Thermo Fisher Scientific, Waltham, MA, USA). Details of the LDTD apparatus were described in previous work [29,59,60,61,62,63,64]. An aliquot of 2 µL of the MMPB extract and an aliquot of 4 µL of the ANA-a extract were individually spotted into a LazWell 96-well sample metal plate. After complete solvent drying at 40 °C for 5 min, the back of the sample well was heated by an infrared laser (980 nm, 20 W, continuous) in the LDTD instrument. The sample was desorbed and thereafter, a carrier gas flow of 2 L∙min^−1^ was set at a temperature of 50 °C to avoid temperature variation and to transfer the gas phase molecules to APCI ionization followed by the HRMS detection. Finally, the optimal laser power was set at 40% and laser pattern was optimized to the following settings for both analytical methods: 2 s at 0%, 2 s ramping from 0% to 40%, 1 s hold at 40%, 0.1 s from 40% to 0% and 2 s hold at 0%, with a total desorption time of 7 s per sample. The APCI ionization was set in negative mode for MCs and in positive mode for ANA-a and was set with these parameters: ion sweep gas 0.3, sheath gas, auxiliary gas, and skimmer offset are set to 0 (all arbitrary values), vaporizer temperature 0 °C, and capillary temperature 350 °C.

#### 4.4.2. UHPLC Conditions

The chromatographic separation was performed with a Thermo Scientific Dionex Ultimate 3000 Series RS pump coupled with a Thermo Scientific Dionex Ultimate 3000 Series TCC-3000RS column compartments and a Thermo Fisher Scientific Ultimate 3000 Series WPS-3000RS autosampler controlled by Chromeleon 7.2 Software (Thermo Fisher Scientific, Waltham, MA, USA and Dionex Softron GMbH Part of Thermo Fisher Scientific, Germering, Germany). The chromatographic column was a Hypersil GOLD^TM^ C18 column (100 mm, 2.1 mm, 1.9 µm particles) preceded by a guard column (5 mm, 2.1 mm, 3 µm particles) (Thermo Fisher Scientific, Waltham, MA, USA). Chromatographic settings are presented in Table 4 for both analytical methods and are based on published optimization parameters [31,33]. For the analysis of MCs and ANA-a, the injection volume was 25 µL and flow rate was set at 525 µL∙min^−1^ with a constant temperature of 55 °C. Concerning the ionization, the final HESI parameters used to maximize signal intensity were as follows: capillary temperature, 350 °C; vaporizer temperature, 450 °C; sheath gas pressure, 35 arbitrary units; aux gas pressure (10 arbitrary units), ion sweep gas pressure, 0 arbitrary units; and spray voltage, +3200 V. For the analysis of CYN, STX and BMAA, the injection volume was 25 µL and flow rate was set at 500 µL∙min^−1^ with a constant temperature of 40 °C. The HESI parameters were as follows: capillary temperature, 400 °C; vaporizer temperature, 350 °C; sheath gas pressure, 30 arbitrary units; aux gas pressure, 10 arbitrary units, ion sweep gas pressure, 0 arbitrary units; and spray voltage, +3000 V. For the analysis of BMAA, as demonstrated in the publication which describes this methodology [33], BMAA is independently detected from two important isobaric isomers, 2,3-diaminobutyric acid and *N*-(2-aminoethyl)-glycine through chromatographic separation and high resolution detection so that the results are not confounded.

#### 4.4.3. HRMS Conditions

The detection was performed using a Q-Exactive mass spectrometer controlled by the Xcalibur 2.3 Software (Thermo Fisher Scientific, Waltham, MA, USA). Instrument calibration in positive and negative modes was done every five days to avoid mass shifts in detection with a direct infusion of a LTQ Velos ESI positive and negative ion calibration solutions (Pierce Biotechnology Inc., Rockford, IL, USA). Targeted ion fragmentation (t-MS^2^) mode was used for quantification and the normalized collision energy (NCE) is presented in Table S3 along with the precursor and product masses of target analytes. The precursor ions are filtered by the quadrupole which operates at an isolation width of 0.4 amu. A resolving power of 17,500 FWHM at *m*/*z* 200 was used with an automatic gain control (AGC) target set at 1 × 10^5^ ions for a maximum injection time of 50 ms. For more technical details, please refer to these previous studies [27,65,66,67].

### 4.5. Method Validation

The data treatment was performed using the Xcalibur 2.3 Software (Thermo Fisher Scientific, Waltham, MA, USA). The mass window applied for the extraction of chromatograms was 5 ppm. The recovery values from the extraction methods and the matrix effects as well as the method validation parameters were evaluated using material consisting of Chlorella-based dietary supplement products. *Chlorella* is a unicellular plant which is not related to cyanobacteria, so it was chosen as matrix blank. Extraction recoveries were determined at two levels depending of the analytical method, see Table 1, using the mean peak area of target compounds spiked prior to extraction in *Chlorella* samples. The results were compared to spiked post-extraction samples and were reported as percentages of recovery. The matrix effects were evaluated with the mean peak area of target compounds in post extraction spiked *Chlorella* samples compared to post extraction spiked matrix-free (dd-H_2_O) samples. They were evaluated at two concentration levels, see Table 1, and were reported as percentages of signal recovery from matrix effects. All recovery values were evaluated using triplicate determinations. Five-point internal calibration curves were prepared in *Chlorella* samples submitted to extraction with concentration levels ranging from 0.03 to 20 µg∙g^−1^ depending on target compounds. For all analytical methods, the concentration of internal standards was selected at 2.5 µg∙g^−1^ for its capacity for signal correction (data not shown). The internal standards were added before extraction steps as follows: 4-phenylbutyric acid (4-PB) for the analysis of MMPB, phenylalanine-D_5_ (PHE-D_5_) for the analysis of ANA-a, nodularin (NOD) for the analysis of MCs and ANA-a and diaminobutyric acid-D_3_ (DAB-D_3_) for the analysis of CYN, STX, and BMAA. Method detection limit (MDL) and method quantification limit (MQL) were determined as 3.3 and 10 times, respectively, the standard deviation of the y-intercept divided by the slope of the calibration curve in *Chlorella* samples. MDL values were confirmed experimentally by analyzing each compound at the statistically calculated concentrations. Accuracy, expressed as relative error (%); and interday/intraday variations, expressed as the relative standard deviation (%), were determined with two different concentration levels on the linearity range (0.5 and 10 µg∙g^−1^, *n* = 3) in *Chlorella* samples. Interday repeatability was estimated over three weeks.

## Figures and Tables

**Table 1:** Method validation parameters including accuracy and precision determined at two different concentrations levels and recovery with standard deviation (STD, *n* = 3).

Compounds ^a^		Accuracy (RE %)		Intraday (RSD %)		Interday (RSD %)		Recovery (%)		*R*^2^	Linearity Range (µg∙g^−1^)	MDL (µg∙g^−1^)	MQL (µg∙g^−1^)
		L	H	L	H	L	H	L	H				
1	MMPB ^b,c^	8	6	7	9	12	12	79 (9)	85 (10)	0.9988	0.6–20	0.2	0.6
	ANA-a ^b^	6	5	6	7	11	13	95 (7)	92 (8)	0.9987	0.8–20	0.3	0.8
2	ANA-a	9	7	5	5	8	9	89 (7)	90 (8)	0.9995	0.1–20	0.04	0.1
	MC-RR	7	8	7	3	11	8	86 (9)	89 (8)	0.9993	0.03–20	0.01	0.03
	MC-YR	8	4	5	2	12	9	85 (7)	90 (6)	0.9992	0.07–20	0.02	0.07
	MC-LR	6	3	7	6	9	7	91 (10)	95 (10)	0.9995	0.06–20	0.02	0.06
	MC-LA	6	4	5	1	9	11	92 (7)	91 (9)	0.9990	0.03–20	0.01	0.03
	MC-LY	7	6	8	5	7	9	87 (8)	92 (8)	0.9995	0.1–20	0.03	0.1
	MC-LW	10	7	9	4	9	10	96 (7)	94 (9)	0.9993	0.05–20	0.02	0.05
	MC-LF	8	5	10	2	11	8	93 (9)	90 (7)	0.9992	0.1–20	0.03	0.1
3	CYN	11	10	11	9	15	12	88 (10)	85 (7)	0.9990	0.1–20	0.04	0.1
	STX	12	10	12	8	13	13	83 (11)	86 (9)	0.9989	0.3–20	0.1	0.3
	BMAA	8	7	8	8	10	12	97 (6)	94 (5)	0.9993	0.08–20	0.02	0.08

^a^ Validation results are divided between the three methods. L (low concentration) and H (high concentrations) are respectively set as the follow: 1 and 10 µg∙g^−1^ (1), 0.1 and 10 µg∙g^−1^ (2), and 0.5 and 10 µg∙g^−1^ (3). ^b^ Analysis using LDTD-APCI-HRMS detection. ^c^ Results reported as total MCs equivalent.

**Table 2:** Cyanotoxins daily intake from CB dietary supplements samples (µg), and comparison with guidelines. Values in brackets represent the detected cyanotoxins as percentages of the WHO adult TDI guideline for MCs and the OHA Public Health Division for ANA-a, assuming the consumption of the maximum dosage and a body weight of 60 kg.

No.	MCs tot ^a,b^	ANA-a ^a^	ANA-a	DH-ANA-a ^c^	E-ANA-a ^c^	MC-RR	MC-YR	MC-LR	MC-LA	MC-LY	MC-LW	MC-LF	CYN	STX	BMAA
1	ND	ND	ND	ND	ND	ND	ND	ND	ND	ND	ND	ND	ND	ND	ND
2	ND	ND	ND	ND	ND	ND	ND	ND	ND	ND	ND	ND	ND	ND	ND
3	ND	ND	ND	ND	ND	ND	ND	ND	ND	ND	ND	ND	ND	ND	ND
4	ND	ND	ND	ND	ND	ND	ND	ND	ND	ND	ND	ND	ND	ND	ND
5	ND	ND	ND	ND	**7.2**	ND	ND	ND	ND	ND	ND	ND	ND	ND	ND
6	ND	ND	ND	ND	ND	ND	ND	ND	ND	ND	ND	ND	ND	ND	ND
7	ND	ND	ND	ND	ND	ND	ND	ND	ND	ND	ND	ND	ND	ND	ND
8	**0.25 (10)**	ND	ND	**0.41**	ND	ND	ND	ND	**0.3 (13)**	ND	ND	ND	ND	ND	ND
9	ND	ND	ND	ND	ND	ND	ND	ND	ND	ND	ND	ND	ND	ND	ND
10	ND	ND	ND	ND	ND	ND	ND	ND	ND	ND	ND	ND	ND	ND	ND
11	**2.5 (104)**	ND	ND	ND	**8.1**	ND	**1.6 (67)**	**0.06 (3)**	**0.08 (3)**	ND	ND	ND	ND	ND	ND
12	ND	ND	ND	ND	ND	ND	ND	ND	ND	ND	ND	ND	ND	ND	ND
13	**0.6 (25)**	ND	ND	ND	ND	ND	ND	**0.03 (1)**	ND	ND	ND	ND	ND	ND	ND
14	ND	ND	ND	ND	ND	ND	ND	ND	ND	ND	ND	ND	ND	ND	ND
15	**16.4 (683)**	ND	**0.30 (5)**	**2.2**	**1.8**	**0.8 (33)**	ND	**8.6 (358)**	**2.2 (92)**	**0.04 (17)**	ND	ND	ND	ND	**0.08**
16	**4.5 (188)**	ND	ND	ND	**0.28**	ND	ND	**3.6 (150)**	**1.2 (50)**	ND	ND	ND	ND	ND	ND
17	**3.3 (138)**	**0.35 (6)**	**0.32 (5)**	**5.8**	ND	ND	**0.18 (8)**	**0.04 (17)**	**2.6 (108)**	ND	ND	ND	ND	ND	**0.44**
18	**0.8 (33)**	ND	ND	ND	ND	ND	ND	**0.52 (22)**	ND	ND	ND	ND	ND	ND	ND

ND—Not detected. ^a^ Analysis using LDTD-APCI-HRMS detection. ^b^ Total microcystins determined via MMPB reported as total MCs equivalent. ^c^ Concentrations are expressed as ANA-a equivalents. ^d^ Total microcystins determined via the summation of individual microcystins measured.

**Table 3:** Information and specifics from cyanobacterial (CB) dietary supplements.

No.	Content	Place of Harvest/Purchased From	Exp. Date	Recommended Maximum Dosage/Day	Specifics
1	*Spirulina*	Pacific/Store	01/2006	4000 mg	—
2	*Spirulina*	NA/Store	02/2006	3000 mg	—
3	*Spirulina*	Hawaii/Store	06/2006	2000 mg	—
4	*Spirulina*	NA/Store	02/2007	2000 mg	—
5	*Spirulina*	NA/Store	08/2007	4500 mg	Not recommended for children
6	*Spirulina*	NA/Store	12/2007	1440 mg	—
7	*Spirulina*	NA/Store	01/2008	3000 mg	Not recommended for children
8	*Spirulina*	Hawaii/Store	06/2008	1200 mg	—
9	*Spirulina*	Hawaii/Store	05/2009	1200 mg	—
10	*Spirulina*	NA/Store	06/2009	3040 mg	Not recommended for children
11	*Spirulina*	NA/Store	07/2015	3000 mg	—
12	*Spirulina*	NA/Internet	05/2015	3000 mg	—
13	*Spirulina*	NA/Internet	06/2015	3000 mg	—
14	*Spirulina*	Hawaii/Internet	06/2015	3000 mg	—
15	*A. flos-aquae*	NA/Store	07/2015	2000 mg	Not recommended for children
16	*A. flos-aquae*	Klamath Lake/Internet	05/2015	3000 mg	Not recommended for children
17	*A. flos-aquae*	Klamath Lake/Internet	06/2015	800 mg	—
18	*A. flos-aquae*	Klamath Lake/Internet	06/2015	1000 mg	—

NA—Not available.

**Table 4:** Gradient parameters used for the analysis of cyanotoxins using UHPLC-HESI-HRMS, where A is HPLC water with 0.1% formic acid, B is acetonitrile with 0.1% formic acid, and C is methanol with 0.1% formic acid.

MCs and ANA-a				CYN, STX and BMAA		
Time (min)	A (%)	B (%)	C (%)	Time (min)	A (%)	B (%)
0.00	75	25	0	0.00	70	30
0.35	45	55	0	2.00	10	90
1.15	45	55	0	4.00	0	100
1.16	55	0	45	6.00	0	100
2.95	55	0	45	6.01	70	30
4.51	5	0	95	10.00	70	30
4.66	75	25	0			
7.00	75	25	0			

## Supplementary Materials

The following are available online at www.mdpi.com/2072-6651/9/3/76/s1, SI1: Chemicals, reagents, and stock solutions, Table S1: Recoveries from matrix effects of targeted cyanotoxins evaluated at two different concentrations levels (µg∙g^−1^) with standard deviation (STD, *n* = 3)., Table S2: Cyanotoxins detection in CB dietary supplements (µg∙g^−1^) with relative standard deviation (RSD-%)., Table S3: Parameters of HRMS detection of target cyanotoxins and internal standards.
