# Supplementary material for: Detection of Cyanotoxins in Algae Dietary Supplements
Source: Toxins (Basel). 2017 Feb 25;9(3):76. doi: 10.3390/toxins9030076 (PMC5371831; doi:10.3390/toxins9030076)
Supplement: Supplementary file 1 [file toxins-09-00076-s001.pdf]

Supplementary Materials: Detection of Cyanotoxins in Algae Dietary Supplements

Audrey Roy-Lachapelle, Morgan Solliec, Maryse F. Bouchard, and Sébastien Sauvé

SI1. Chemicals, reagents and stock solutions

2-methyl-3-methoxy-4-phenylbutyric acid sodium salt (MMPB, purity ≥ 94%) was purchased from Wako Pure Chemical Industries, Ltd. (Osaka, Japan). 4-phenylbutyric acid (4-PB, purity ≥ 99%), L-BMAA hydrochloride (BMAA, purity ≥ 97%), DL-phenylalanine (PHE, purity ≥ 99%) and nodularin (NOD, ≥ 99%) were obtained from Sigma-Aldrich (Oakville, ON, Canada). All selected standards for microcystins (MCs-RR, -YR, -LR, -LY, -LW, -LF and –LA, purity ≥ 99%), anatoxin-a (ANA-a, purity ≥ 99%) and cylindrospermopsin (CYN, purity ≥ 99%) were purchased from Abcam Biochemicals (Cambridge, MA, USA). 2,4 diaminobutyric acid-2,4,4-D_3 dihydrochloride (DAB-D_3, 99 atom % D) and DL-phenylalanine ring-D_5 (PHE-D_5, purity ≥ 98%) were purchased from CDN isotopes (Pointe-Claire, QC, Canada). Ampoule of certified standard solutions of saxitoxin dihydrochloride (STX, 66.3 μM in 3 mM hydrochloric acid) was obtained from the Certified Reference Materials Program (NRC, Halifax, NS, Canada). Potassium permanganate ($KMnO_4$, purity ≥ 99,0%), sodium (meta)periodate ($NaIO_4$, purity ≥ 99,0%), sodium bisulfate (purity ≥ 95%), sodium hydroxide (NaOH, purity ≥ 98%), sodium tetraborate (Borax, purity ≥ 99%), dansyl chloride (DNS, purity ≥ 99%), citric acid (purity ≥ 99.5%), and formic acid (HCOOH, purity ≥ 95.0%), hydrochloric acid (HCl, ACS reagent 37%) and sulfuric acid standard solution (1.000 M) were obtained from Sigma-Aldrich (Oakville, ON, Canada). All solvents used were of high performance liquid chromatography (HPLC) grade purity from Fisher Scientific (Whitby, ON, Canada). Deionized/distilled water (dd-H_2O) was used recovery evaluation. Compressed air (Ultra Zero Certified grade; ≤ 2 ppm water) used as carrier gas for the LDTD desorption was purchased from MEGS Specialty Gases, Inc. (St-Laurent, QC, Canada).

Table S1. Recoveries from matrix effects of targeted cyanotoxins evaluated at two different concentrations levels (μg g^{-1}) with standard deviation (STD, n = 3).

Compounds [a]		Signal Recovery from Matrix Effect ± STD (%)	
		Low	High
1	MMPB [b,c]	85 ± 11	87 ± 8
	ANA-a[b]	90 ± 10	92 ± 9
2	ANA-a	93 ± 5	95 ± 6
	MC-RR	91 ± 7	94 ± 5
	MC-YR	94 ± 4	92 ± 5
	MC-LR	98 ± 5	102 ± 7
	MC-LA	109 ± 8	105 ± 6
	MC-LY	95 ± 3	91 ± 6
	MC-LW	103 ± 6	108 ± 8
	MC-LF	112 ± 9	104 ± 8
3	CYN	108 ± 10	102 ± 7
	STX	94 ± 8	98 ± 9
	BMAA	105 ± 4	109 ± 6

[a] Validation results are divided between the three methods. Low concentration and High concentrations are respectively set as the follow: 1 and 10 μg g^{-1} (1), 0.1 and 10 μg g^{-1} (2) and 0.5 and 10 μg g^{-1} (3). [b] Analysis using LDTD-APCI-HRMS detection. [c] Results reported as total MCs equivalent.

Table S2. Cyanotoxins detection in CB dietary supplements (μg g^{-1}) with relative standard deviation (RSD-%).

No.	MCs tot [a,b]	ANA-a[a]	ANA-a	DH-ANA-a[c]	E-ANA-a[c]	MC-RR	MC-YR	MC-LR	MC-LA	MC-LY	MC-LW	MC-LF	MCs tot[d]	CYN	STX	BMAA
1	ND	ND	ND	ND	ND	ND	ND	ND	ND	ND	ND	ND	ND	ND	ND	ND
2	ND	ND	ND	ND	ND	ND	ND	ND	ND	ND	ND	ND	ND	ND	ND	ND
3	ND	ND	ND	ND	ND	ND	ND	ND	ND	ND	ND	ND	ND	ND	ND	ND
4	ND	ND	ND	ND	ND	ND	ND	ND	ND	ND	ND	ND	ND	ND	ND	ND
5	ND	ND	ND	ND	**1.6 (12)**	ND	ND	ND	ND	ND	ND	ND	ND	ND	ND	ND
6	ND	ND	ND	ND	ND	ND	ND	ND	ND	ND	ND	ND	ND	ND	ND	ND
7	ND	ND	ND	ND	ND	ND	ND	ND	ND	ND	ND	ND	ND	ND	ND	ND
8	**0.21(11)**	ND	ND	**0.34 (12)**	ND	ND	ND	ND	**0.25 (9)**	ND	ND	ND	**0.25**	ND	ND	ND
9	ND	ND	ND	ND	ND	ND	ND	ND	ND	ND	ND	ND	ND	ND	ND	ND
10	ND	ND	ND	ND	ND	ND	ND	ND	ND	ND	ND	ND	ND	ND	ND	ND
11	**0.84 (12)**	ND	ND	ND	**2.7 (11)**	ND	**0.53 (7)**	**0.02 (5)**	**0.08 (6)**	ND	ND	ND	**0.63**	ND	ND	ND
12	ND	ND	ND	ND	ND	ND	ND	ND	ND	ND	ND	ND	ND	ND	ND	ND
13	**0.24 (8)**	ND	ND	ND	ND	ND	ND	**0.01 (11)**	ND	ND	ND	ND	**0.01**	ND	ND	ND
14	ND	ND	ND	ND	ND	ND	ND	ND	ND	ND	ND	ND	ND	ND	ND	ND
15	**8.2 (7)**	ND	**0.15 (7)**	**1.1 (13)**	**0.92 (10)**	**0.4 (9)**	ND	**4.3 (7)**	**1.1 (4)**	**0.02 (8)**	ND	ND	**5.8**	ND	ND	**0.04 (8)**
16	**1.5 (11)**	ND	ND	ND	**0.07 (9)**	ND	ND	**1.2 (5)**	**0.4 (6)**	ND	ND	ND	**1.6**	ND	ND	ND
17	**4.1 (9)**	**0.44 (9)**	**0.40 (9)**	**7.2 (12)**	ND	ND	**0.22 (6)**	**0.05 (9)**	**3.2 (4)**	ND	ND	ND	**3.4**	ND	ND	**0.55 (5)**
18	**0.8 (8)**	ND	ND	ND	ND	ND	ND	**0.52 (7)**	ND	ND	ND	ND	**0.52**	ND	ND	ND

ND – Not detected. [a] Analysis using LDTD-APCI-HRMS detection. [b] Total microcystins determined via MMPB reported as total MCs equivalent. [c] Concentrations are expressed as ANA-a equivalents.

Table S3. Parameters of HRMS detection of target cyanotoxins and internal standards.

**Compounds**	**RT (min)**	**Experimental precursor mass (*m*/*z*)**	**Quantification fragment (*m*/*z*)**	**Confirmation fragment (*m*/*z*)**	**NCE [b] (%)**
MMPB[a]	NA	207.1019	135.0808	91.0547	30
4-PB[a]	NA	163.0756	91.0546	NA	35
ANA-a[a]	NA	166.1231	149.0965	131.0859	30
PHE-D_5[a]	NA	171.1181	154.0917	NA	30
ANA-a	0.96	166.1231	149.0965	131.0859	30
DH-ANA-a	1.01	168.1383	140.1069	164.1068	30
E-ANA-a	1.04	182.1176	133.1010	93.0696	30
MC-RR	2.42	519.7907	135.0804	103.0542	35
MC-YR	2.61	1045.5353	135.0804	213.0870	45
MC-LR	2.68	995.5560	135.0804	213.0870	40
MC-LA	3.43	910.4920	135.0804	213.0870	35
MC-LY	3.77	1002.5188	265.1587	135.0804	40
MC-LW	4.24	1025.5347	891.5199	213.0870	35
MC-LF	4.49	986.5239	213.0870	375.2391	35
NOD	2.45	825.4505	103.0542	NA	45
CYN	1.26	649.1744	194.1291	176.1184	35
STX	2.97	533.1925	204.0877	138.0665	35
BMAA	3.65	585.1836	277.1007	71.0131	30
DAB-D_3	3.50	588.2024	279.1130	NA	25

NA – Not applicable. [a] Analysis using LDTD-APCI-HRMS detection. [b] Fragmentation energy for precursor ion in HCD cell.
